# Structural Equation Modeling to Evaluate the Effect of Body Shape Concern and Body Appreciation on Eating Behavior Pattern among University Students

**DOI:** 10.18502/ijph.v49i10.4710

**Published:** 2020-10

**Authors:** Maryam KHEIROLLAHPOUR, Mahmoud DANAEE, Amir Feisal MERICAN, Asma Ahmad SHARIFF

**Affiliations:** 1. Institute of Graduate Studies (IGS), University of Malaya, Kuala Lumpur, Malaysia; 2. Academic Development Centre (ADEC), Deputy Vice Chancellor (Academic & International) Office, University of Malaya, Kuala Lumpur, Malaysia; 3. Institute of Biological Sciences, Faculty of Science, University of Malaya, Kuala Lumpur, Malaysia; 4. Center of Research for Computational Sciences and Informatics in Biology, Bio Industry, Environment, Agriculture and Healthcare (CRYSTAL), University of Malaya, Kuala Lumpur, Malaysia; 5. Mathematics Division, Centre for Foundation Studies in Science (ASASI), University of Malaya, 50603 Kuala Lumpur, Malaysia

## Dear Editor-in-Chief

Since body shape concern (BSC) and body appreciation (BA) are known as two influential factors on eating behavior patterns this study aimed to investigate the association of these factors and eight categories of eating behavior patterns. Therefore, structural equation modeling (SEM) was applied to examine the relationship among these factors. Through multistage random sampling, 440 students of University of Malaya (230 males and 210 females) were chosen.

Eating behavior pattern questionnaire (EBPQ) is the popular tools to evaluate the determinants of eating behavior. EBPQ consists of eight categories, including low-fat eating, healthy eating, planning for food, eating outside, meal skipping, snacking, sweets, and emotional eating (1). The first three categories exhibit the healthy eating behaviors, while the rest show the unhealthy eating behaviors. Body shape concern questionnaires (BSCQ) is another questionnaire that comprising the comparative perception, the behaviors adopted as a result of changes in body image and the perception of drastic changes in body image (2). Also, body appreciation scale (BAS) validated by Avalos et al (3) measures the positive perception an individual has on their own body.

**Fig. 1:**
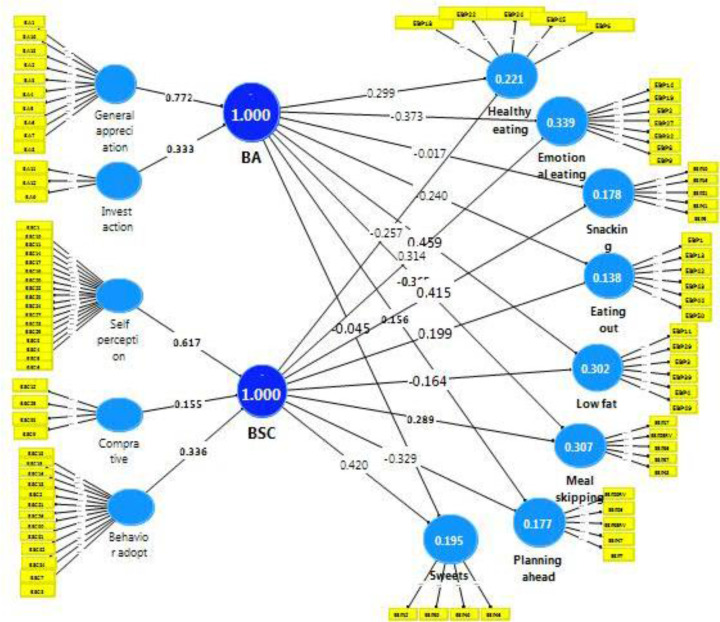
Structural Model

Using SEM analysis, after verifying the convergent and discriminant validity, the association of these factors were identified through path model and structural model. Accordingly, 16 paths; which were linked to causal relationships of BA and BSC (two factors) and eight categories of EBP (Fig. 1), were verified. The results indicated that the effect of body shape concern on the subscales of eating behavior patterns is significant (*P* = 0.01), as an increase in BSC directly causes a proportionate increase in unhealthy eating patterns especially in the subscales of eating outside, emotional eating, meal skipping, snacking and sweets. It can be seen that the effect of the BSC on snacking was the greatest, followed by emotional eating, eating out, meal skipping, and finally sweets. Similar results were obtained in study of body shape concerns and related eating behaviors among Indian urban adolescent girls (4).

However, the BSC had an inverse effect on lowfat eating; as a healthy eating pattern. This means that an increasing BSC resulted in a decrease in low-fat eating (5). Which indicated that unhealthy eating behaviors were associated with body shape concern.

On the other hand, our study showed that body shape concern was not significantly associated with healthy eating (6). This study found no significant relationship between planning for food and body shape concern among youth. However, the body shape and weight concerning adolescents were significantly associated with planning for food, snacking, and meal skipping (7). These results support our findings which showed that an increase in body shape concern was related to an increase in meal skipping and snacking among the youth.

Furthermore, our study showed that an increasing body appreciation results in an increase in healthy eating, low-fat eating, and planning for food, whereas the same increase in body appreciation resulted in a decrease in eating outside, emotional eating, meal skipping, snacking and sweets. The results showed that an increasing body appreciation will increase planning for food and low-fat eating. On the other hand, a higher body appreciation had an inverse effect on meal skipping, eating outside, snacking and sweets. Consistent with previous research, body appreciation may encourage healthy eating behaviors (8).

To the best of researchers’ knowledge, there is no study highlighting the body appreciation’s contribution to eating behavior patterns in among university students. Our work demonstrated that body appreciation was an influential variable with a significant contribution to unhealthy and healthy eating patterns.

## Ethical Consideration

Ethics approval was sought for this study through the relevant university Ethics Committee, (UM.TNC2/RC/H&E/UMREC-63).
